# “I didn't want to do it!” The detection of past intentions

**DOI:** 10.3389/fnhum.2015.00608

**Published:** 2015-11-05

**Authors:** Andrea Zangrossi, Sara Agosta, Gessica Cervesato, Federica Tessarotto, Giuseppe Sartori

**Affiliations:** ^1^Department of General Psychology, University of PaduaPadua, Italy; ^2^Italian Institute of Technology, Center for Neuroscience and Cognitive SystemsRovereto, Italy

**Keywords:** past intentions, reaction times, mens rea, autobiographical Implicit Association Test, voluntary manslaughter

## Abstract

In daily life and in courtrooms, people regularly analyze the minds of others to understand intentions. Specifically, the detection of intentions behind prior events is one of the main issues dealt with in courtrooms. To our knowledge, there are no experimental works focused on the use of memory detection techniques to detect past intentions. This study aims at investigating whether reaction times (RTs) could be used for this purpose, by evaluating the accuracy of the autobiographical Implicit Association Test (aIAT) in the detection of past intentions. Sixty healthy volunteers took part in the experiment (mean age: 36.5 y; range: 18–55; 30 males). Participants were asked to recall and report information about a meeting with a person that had occurred at least 1 month before. Half of the participants were required to report about an intentional meeting, whereas the other half reported on a chance meeting. Based on the conveyed information, participants performed a tailored aIAT in which they had to categorize real reported information contrasted with counterfeit information. Results demonstrated that RTs can be a useful measure for the detection of past intentions and that aIAT can detect real past intentions with an accuracy of 95%.

## Introduction

“*Andreas Lubitz was alone in the cockpit, breathing in silence, as his captain pounded on a locked door and passengers screamed. Those chilling sounds—captured in a black-box recording—have left French investigators with little doubt that the crash that killed 150 people aboard Flight 9525 was deliberate*.” These are the words used by *The Wall Street Journal* on March 26th, 2015 to describe the tragic plane crash of a few days before.

The ability to detect intentions takes on great importance for investigators and intelligence, as it deals with the issue of preventing criminal acts from occurring (Vrij et al., 2011). In the research field, intention is defined as an actor's mental state preceding a corresponding action, usually coming with a strong commitment to perform the intended action (Malle et al., 2001) and often accompanied by a degree of planning (Granhag and Mac Giolla, 2014). Searle (1983) refers to these deliberations of a future action as “prior intentions” and as the initial representation of the goal of an action prior to the initiation of the action. These definitions presume that prior intentions are not just derived from a vague willpower concerning the actor's life but are also targeted and purpose-driven, associated with a planning of the necessary actions to reach the end goal. Moreover, some researchers argue that there are different types of intentions from a temporal point of view and suggest a possible distinction between long-term antecedents of action (prior intentions; Searle, 1983) and short-term antecedents of actions (intentions in action; Searle, 1983; Becchio et al., 2008a,b; Sartori et al., 2009). In some legal systems, such as in the United States, the concept of intentionality has many synonyms including *voluntarily, purposely, knowingly*, and *willfully* (Malle and Nelson, 2003). Thus, the concept of “intention” as antecedent of action is strongly related to those of volition, free will, and responsibility. In the legal context, decisions are based on the assumption that human behavior is driven by intentions; therefore, individuals are considered responsible for their voluntary actions insofar as they behave on the basis of their own free will.

In cognitive neuroscience, this has been a challenging issue since the first experiment by Libet et al. (1983). These authors found that the Readiness Potential (RP) precedes the awareness of the intention to act by 300–800 ms, demonstrating that subjective awareness of a voluntary action occurs after the ultimate initiation of the act (Libet et al., 1983). According to Hallett (2007), under this perspective, intention could be considered as a perception of a motor state that has already been determined in the brain. Starting from this standpoint, a number of independent research groups found that the conscious intention is preceded by unconscious brain activity in the motor areas (e.g., Haggard and Eimer, 1999; Rigoni et al., 2013) and that through this unaware activity it is possible to predict the outcome of a decision up to several seconds before it enters awareness (e.g., Soon et al., 2008, 2013). Moreover, some research focused on the detection of prior intentions, distinguishing them from lies. For instance, in one of the first experiments on lying about intentions, Vrij et al. (2011) asked passengers in an airport departure hall to tell the truth or to lie about their forthcoming trip. The authors found that, analyzing the plausibility of the answers to an interview, the accuracy in the detection of truth tellers and liars was 72 and 74%, respectively (Vrij et al., 2011). In another study (Agosta et al., 2011a), researchers went a step further, investigating whether real and false intentions could be distinguished through reaction times (RTs), and they found an accuracy of 100% in discriminating prior intentions from plausible but false intentions and from the subject's hopes and expectations.

Taken together, these data suggest that real intentions are detectable through cognitive and behavioral cues.

Unfortunately, in almost all criminal cases, such as the plane crash of March 2015 mentioned above, society has to deal with the impossibility of detecting intentions before a crime happens. Thus, the focus must move from the ideal goal of crime prevention to a better understanding of crimes after they happen. In daily life and in courtrooms, people regularly analyze the goings-on in the minds of others. For thousands of years, moral and legal systems have encompassed concepts such as intention, motive, and forethought when debating their proper definition and their relation to responsibility, blame, and punishment (Malle and Nelson, 2003). In modern society, too, although growing importance is given to computers and machines, no one would accept as fair and legitimate a legal system that avoids considerations of an agent's mental state when committing a crime. For this reason, in criminal law, judges are requested to enter the criminal's mind in order to reach as thorough as possible an understanding of his intentions and thus to choose an appropriate punishment. Basically, four conditions have to be satisfied to establish criminal responsibility: (a) the defendant must have committed an act which is considered a crime (this is called the *actus reus* in common law systems); (b) that act must have been committed in a specific “state of mind” (this is known as the *mens rea* in common law systems); (c) there must be a causal connection between the crime and the prohibited consequences; and (d) there must be an absence of circumstances that would constitute a legal defense to any crime charged (Carson and Felthous, 2003).

Therefore, considering the above-mentioned legal framework, we can state that in this study we face the concept of *mens rea* from a neuroscientific point of view. The concept of *mens rea* refers to the intent of committing the offense; it is the mental element of an offense, including the awareness by a person that his or her own conduct is criminal. Under this perspective, detection of intentions behind an occurred event (crime) is one of the main issues dealt with in courtrooms. Nevertheless, this target is pursued basically through an inferential process based on behavioral and circumstantial evidence, usually without considering a scientific approach to autobiographical memory as the key for detecting past intentions. Some research suggests that a range of physiological (e.g., see Meijer et al., 2014), psychophysiological (e.g., Winograd and Rosenfeld, 2010; Nahari and Ben-Shakhar, 2011), neural (e.g., Rissman et al., 2010), and cognitive (e.g., Sartori et al., 2008) parameters might be used to detect memories. The so-called Memory Detection refers to a family of techniques using indirect measures to detect memories (e.g., a real autobiographical memory) among alternatives. Basically, whereas the goal of lie detection is to identify whether a response is a truthful response or a lie, the target of memory detection is to establish whether a specific event is represented in a subject's autobiographical memory. In general, this methodology is based on the comparison between critical information (included in a specific autobiographical memory) together with similar, but not critical, information (plausible, but not part of that memory). One of the potentially most efficient cognitive measures for memory detection is represented by RTs (e.g., Seymour et al., 2000; Seymour and Kerlin, 2008).

To our knowledge there are no experimental works focused on the use of memory detection techniques to detect past intentions, except for a study focused on the detection of reasons for producing so-called “white lies” (Agosta et al., 2013), a subtype of lies which most people tell on a daily basis in order to place themselves or others in a more positive light (Granhag and Vrij, 2005). These authors tested the accuracy of the autobiographical Implicit Association Test (aIAT; Sartori et al., 2008), a memory detection tool based on the analysis of a subject's RTs, in detecting reasons underlying white lies. The main results indicate that aIAT can accurately discriminate real reasons underlying white lies in 95% of cases.

Here we present an experiment aimed at evaluating whether past intentions may be identified using the aIAT.

The aIAT (Sartori et al., 2008) is a novel variant of the Implicit Association Test (Greenwald et al., 1998) that can be used to establish whether an autobiographical memory trace is encoded within the respondent's mind. More specifically, with the aIAT, it is possible to evaluate which one of two autobiographical events is true (Sartori et al., 2008). Nevertheless, aIAT has been tested in different domains and on different constructs such as future, medium, and long-term intentions (Agosta et al., 2011a), white lies and underlying intentions (Agosta et al., 2013), mock crimes (Sartori et al., 2008), holidays (Sartori et al., 2008), cocaine/heroine consumption (Sartori et al., 2008), driver's licenses (Sartori et al., 2008), flashbulb memories (e.g., Curci et al., 2015), and whiplash malingering (Sartori et al., 2007). Thus, the aIAT has been validated in both forensic and clinical settings. It has been demonstrated that this tool can determine which of two autobiographical events is true with 91% accuracy (Sartori et al., 2008). Despite the different kinds of investigations and constructs aIAT has been applied to (see Agosta and Sartori, 2013, for a review), the method's structure is always maintained.

The aIAT includes stimuli belonging to four categories: two of them are logical categories represented by sentences that are certainly true (e.g., “I am in front of a computer”) or certainly false (e.g., “I am climbing a mountain”) for the respondent and relate to the moment of testing. The other two categories are represented by alternative versions of the construct under investigation (e.g., “I went to Paris for Christmas” vs. “I went to London for Christmas”) only one of the two being true. The task is basically a categorization task. The basic principle of aIAT is that the pairing of sentences about a truly autobiographical event with certainly true sentences should facilitate faster responses, so that the specific pattern of RTs for the double categorization blocks indicates which autobiographical event is either true or false. The true autobiographical event is identified because it determines faster RTs when sharing the same motor response with certainly true sentences.

The aIAT is structured in five classification blocks: three simple categorization blocks (1, 2, 4), and two combined categorization blocks (3 and 5). In simple blocks, each response button is used to classify sentences related to only one category. In double blocks, each response button is used to classify sentences related to two different categories. In Block 1, participants have to classify true and false sentences (e.g., I am in front of a computer vs. I am in front of a television) using two response keys, one on the left and one on the right of the keyboard. In Block 2, participants have to classify autobiographical sentences (e.g., I went to Paris for Christmas vs. I went to New York for Christmas) with the same two response keys. In Block 3 (double categorization block), true sentences and sentences related to the first autobiographical event (e.g., Christmas in Paris) are paired on the same response key and false sentences and sentences related to the second autobiographical event (e.g., Christmas in New York) are classified with the other response key. In Block 4, only autobiographical events are reverse classified with the two response keys. Finally, in Block 5, participants have to classify both true sentences and sentences related to the second autobiographical event (Christmas in New York) with the same response key, and false sentences and the first autobiographical event (Christmas in Paris) with the other key.

As already noted, aIAT is a flexible tool and has been applied to different constructs. This is possible because one of the main features of aIAT is that items are presented as sentences. For this reason, aIAT can be considered as an adequate tool to describe intentions in full.

In previous works, aIAT was used to detect prior intentions about future actions (Agosta et al., 2011a) and reasons underlying previously told white lies (Agosta et al., 2013), but it was never applied to the detection of intentions behind a past autobiographical event. Here we present an experiment aimed at evaluating whether past intentions may be identified using the aIAT (Sartori et al., 2008). We investigated past intentions by asking subjects to recall and describe an accidental meeting or an intentionally planned one.

## Methods

### Participants

Sixty healthy volunteers took part in the experiment (mean age: 36.5 y; range: 18–55; 30 males). All of them gave a signed informed consent, and the project has been approved by the Ethical Committee for the Psychological Research of the University of Padua. Participants were subdivided in two groups: the “chance” group and the “intentional” group, on the basis of the intentionality underlying a reported meeting with a person at least 1 month before. Each group was composed of 30 subjects (15 males).

### Materials and procedure

In Phase 1, participants were asked to recall a meeting with a person that occurred at least 1 month before and to fill out a form reporting its main features. Participants in the chance group were requested to report a meeting that happened by chance; in particular, they were asked to specify (a) who they met (his/her name and role in their life), (b) when the meeting happened, (c) what they were doing, and (d) where they were at that moment. By contrast, participants in the intentional group were requested to report an intentional and planned meeting specifying (a) who they decided to meet, (b) how they organized the meeting, (c) why they wanted to meet that person, and (d) at what time and (e) where they decided to meet. Moreover, participants in the intentional group were told that they should have directly organized the selected meeting and that it should not have been a routine meeting. After that, starting from the specific information provided by each participant through the form, we built sentences about the real intentionality underlying the meeting (intentionally organized vs. happened by chance) and sentences describing the opposite scenario.

An example of a sentence about real intentions for participants in the chance group was “I met Andrew by chance,” while an opposite sentence for the same participant was “I decided to meet Andrew.” In the same way, sentences for the intentional group described their meeting planning and an unreal scenario in which they met the person by chance. In Phase 2, these sentences were used to build a tailored aIAT for each participant. The participants' task on the aIAT was to logically categorize true and false statements related to the moment of testing (taken from previous research: e.g., “I'm in front of a computer”) and statements about real and counterfeit intentions related to the meeting reported in Phase 1 (into categories “Chance” vs. “Intentional”; see Table 1 for a sample list of sentences used in the experiment). Each of the four categories (true vs. false and Chance vs. Intentional) was represented by five statements.

**Table 1:** **An example of a sentences' list used in the experiment**.

**Labels**	**Italian sentences**	**English translation**
True	1. Sono di fronte ad un computer	1. I'm in front of a computer
	2. Sto rispondendo con la tastiera	2. I'm answering with the keyboard
	3. Sono seduto sulla sedia	3. I'm sitting on a chair
	4. Sto facendo un test di Psicologia	4. I'm doing a psychological test
	5. Sono in università	5. I'm inside the university building
False	1. Sono di fronte ad un televisore	1. I'm in front of the television
	2. Sto rispondendo con la matita	2. I'm answering with the pencil
	3. Sono seduto sulla panchina	3. I' sitting on a bench
	4. Sto facendo un test di matematica	4. I'm doing a mathematical test
	5. Sono in ospedale	5. I'm inside the hospital building
Chance	1. Ho incontrato Andrea per caso	1. I met Andrew by chance
	2. Ho visto Andrea senza prevederlo	2. I met Andrew without predict it
	3. Ho incrociato Andrea per strada	3. I met and crossed Andrew on the road
	4. Ho trovato Andrea senza volerlo	4. I didn't want to came across Andrew
	5. Mi sono imbattuta nel mio ex compagno	5. I did chance upon my ex-boyfriend
Intentional	1. Ho deciso di trovarmi con Andrea	1. I decided to come across Andrew
	2. Ho sentito Andrea per trovarci	2. I got in touch with Andrew to meet him
	3. Ho deciso di incontrare Andrea	3. I planned to meet Andrew
	4. Volevo vedere Andrea lungo il fiume	4. I wanted to see Andrew on the river
	5. Ho voluto vedere il mio ex compagno	5. I desired to meet my ex-boyfriend

*This is an example of sentences' list coming from a participant in the chance group*.

**Figure 1:**
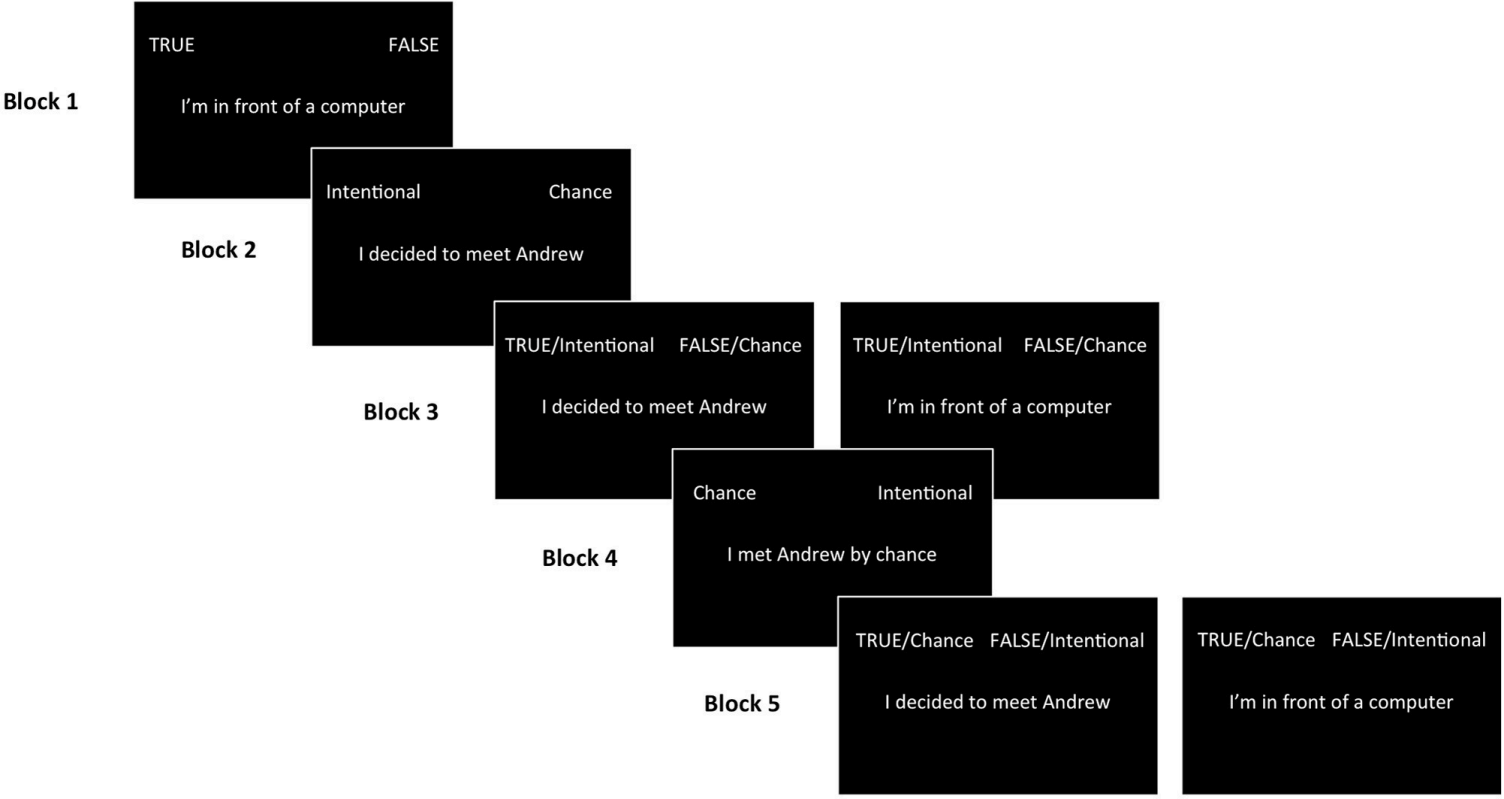
**An example of the experimental procedure of the aIAT built for an intentional group participant**. Participants were asked to classify the stimulus (i.e., the sentence displayed) as fast and accurately as possible by pressing the left (i.e., “A” key) or the right (i.e., “L” key) key. In Block 3 (congruent block) logically true sentences and stimuli describing real information (about the intentionality behind the reported meeting) shared the same response key (i.e., the left key). In Block 5 (incongruent block) the left response key was shared by true sentences and stimuli describing counterfeit information.

Participants completed five separate blocks of categorization trials, as expected by the previously discussed standard aIAT structure (as shown in Figure 1).

For each trial, the stimulus sentence was presented in the center of the screen. The participants were requested to classify the sentence as quickly and accurately as possible by pressing one of two keys, one on the right (i.e., key L) and one on the left (i.e., key A) of a keyboard. Greenwald and Nosek (2001) reported no effects on IAT scores of subject self-reported handedness. In Block 1 (20 trials), participants classified stimuli along the logical dimension (true vs. false) by pressing the left key if the sentence was true (e.g., “I'm in front of a computer”) or the right key if the sentence was false (e.g., “I'm in front of a television”). In Block 2 (20 trials), participants classified sentences along the critical dimension: Intentional vs. Chance. They were asked to press the left key to classify sentences expressing the real information they reported (for an intentional group participant, e.g., “I decided to meet Andrew”) and to press the right key to classify sentences expressing counterfeit information (for an intentional group participant, e.g., “I met Andrew by chance”). In Block 3 (60 trials, double categorization block), true sentences and sentences describing real participants' information were associated (“congruent” block). Participants were requested to press the left key if the sentence was either true or indicated the real information they gave and the right key if the sentence was false or indicated counterfeit information (e.g., an intentional group participant pressed the left key for true and for “Intentional” sentences and the right key for false and for “Chance” sentences). In Block 4 (40 trials), participants were requested to perform a reversed classification of Block 2: they pressed the left key for counterfeit information sentences and the right key for real information sentences. In Block 5 (60 trials, double categorization block), true sentences and sentences describing counterfeit participants' information were associated (“incongruent” block). Participants were asked to press the left key for true sentences and sentences about counterfeit information, and the right key for false sentences and sentences about real information (e.g., an intentional group participant pressed the left key for true and for “Chance” sentences and the right key for false and for “Intentional” sentences).

Labels indicating category names were displayed on the computer screen for the entire duration of the experiment. Previous works (Agosta et al., 2011c, 2013) showed that when comparing a direct version (with Block 3 as congruent and Block 5 as incongruent) and a reversed version (Block 3 as incongruent and Block 5 as congruent) of aIAT, there was no reduction in accuracy for the identification of the true autobiographical memory (Agosta and Sartori, 2013); thus, in this study only the direct version was administered.

The comparison between RTs in congruent and incongruent blocks is the key point for the aIAT data analysis (Sartori et al., 2008). Blocks 1, 2, and 4 are considered as training blocks and they are not contemplated in the analysis.

### Dependent measures

Two dependent measures were considered: RTs for the double categorization blocks (Blocks 3 and 5) and the D-IAT index (Greenwald et al., 2003) calculated for each participant independently. Before any further analysis, RTs shorter than 150 ms or longer than 10,000 ms were discarded; moreover, RTs for incorrect responses were replaced with the mean of correct latencies plus 600 ms. The D-IAT index was calculated by subtracting the average RTs of the congruent block from the average RTs of the incongruent block and then dividing this difference by the “inclusive” standard deviation of subject response latencies in the two combined blocks (Greenwald et al., 2003). As this index is grounded on the difference between incongruent and congruent blocks, it results in a positive value if response latencies are faster in the congruent than in the incongruent block; otherwise the value is negative. Therefore, this indicates an association between the categories sharing the same motor response in the congruent block (Greenwald et al., 1998; Sartori et al., 2008). In our experiment, the positive value of a participant's D-IAT states that there is an implicit association between true statements and sentences about the real degree of intentionality, leading to a correct classification. Greenwald et al. (2003) argued that in a sense, D-IAT can be seen as an effect size measure, as the division of a difference between means by a standard deviation is similar to one of the most known effect size measures, Cohen's *d* (Cohen, 1977). The distinction is that the standard deviation in the D-IAT is computed from the scores in both blocks, ignoring the condition (Greenwald et al., 2003).

### Data analysis

Given the structure of the RTs data, we used a generalized linear mixed-effect model approach (Pinheiro and Bates, 2000) to investigate whether Congruency (congruent vs. incongruent) had an effect on response latencies in a group analysis. This statistical approach has been applied in many research areas (e.g., Goldstein, 2005; Faraway, 2006; Malcolm et al., 2008; Levitan et al., 2015), and recently it has been suggested for the analysis of RTs (Baayen and Milin, 2010), as it is considered an effective method in dealing with complex data. The use of mixed-effect models entails several advantages, in particular they allow the simultaneous consideration of all factors potentially contributing to the data understanding (Baayen et al., 2008). These factors include not only the fixed effect factors (controlled by the experimenter) but also so-called random effects factors (e.g., participants), characterized by the fact that their levels are randomly drawn from a population (Di Giorgio et al., 2012).

In the present study, mixed-effects models were adopted not only to deal with the skewness of data distribution, but for two main reasons specifically linked to aIAT features: (a) to include repeated measures in the model, without the need to average across trials and thus enhancing statistical power; and (b) to model participants' individual differences in RTs distribution as random effect.

Three Generalized Linear Mixed-Effects regression models with maximum likelihood estimation were built, with reaction-times as dependent variable, subject as the random effect factor and Congruency (congruent vs. incongruent) and Group (chance vs. intentional) as fixed effects regressors. The null model—model 0—contained random effect only. Model 1 included Congruency as predictor, and in Model 2 the contribution of Group was added. We chose to use a Gamma-family function as link function as it is considered a good model for approximating RTs (Whelan, 2008; Baayen and Milin, 2010). Models were then compared through the Akaike Information Criterion (AIC; Akaike, 1987) and the Bayesian Information Criterion (BIC; Schwarz, 1978).

Because we used a Gamma-family link function to deal with the non-normality of RTs data, the β regression coefficients estimate of our best model was not directly interpretable in a quantitative sense. Thus, in order to reach a better understanding of the Congruency effect on RTs, we back-transformed the corresponding β coefficient. In particular, we applied the inverse of the Gamma link function and we calculated the difference between incongruent and congruent block (ICDiff) as follows:

$$\begin{array}{l} \text{ICDiff}=\frac{1}{\alpha +\beta }-\frac{1}{\alpha } \end{array}$$

where α was the model intercept and β was the Congruency coefficient.

For the D-IAT analysis, first we calculated a specific D-IAT for each participant to examine the aIAT's accuracy, as discussed above. Then a linear regression model with only Group variable as predictor was built to investigate whether there was a difference in D-IATs between groups. Congruency was not considered in the model because D-IAT itself expresses the congruency effect.

All analyses were performed using R software (R Core Team, 2013). For generalized linear mixed-effect models, we used the R package lme4 (Bates and Maechler, 2010). Results for each dependent variable are presented in the following section.

## Results

The model comparison showed that both AIC and BIC decreased from 107,985 and 108,005, respectively, for the model with only the random effect included (Model 0) to 107,328 and 107,355, respectively, for the model with the Congruency predictor added (Model 1), resulting in the best model to explain the data distribution (Table 2). The addition of the Group variable (Model 2) did not significantly contribute toward explaining the data, as AIC remained the same while BIC was higher (107,362) indicating the most parsimonious model—Model 1—as the better one. This was confirmed through a Likelihood Ratio Test (LR) which showed that Model 1 was significantly better than Model 0 [$\chi_{\left(4\right)}^{2}=658.7$, *p* < 0.001] and that Model 2 was not significantly better than Model 1 (*p* = 0.11) in explaining data.

**Table 2:** **Model comparison**.

**Dependent variable: RTs**		
**Model**	**AIC**	**BIC**
Model 0: random effect	107,985	108,005
Model 1: random effect + Congruency	107,328	107,355
Model 2: random effect + Congruency + Group	107,328	107,362

*Random and fixed effect factors predicting RTs. RTs, reaction-times; AIC, Akaike Information Criterion; BIC, Bayesian Information Criterion*.

**Figure 2:**
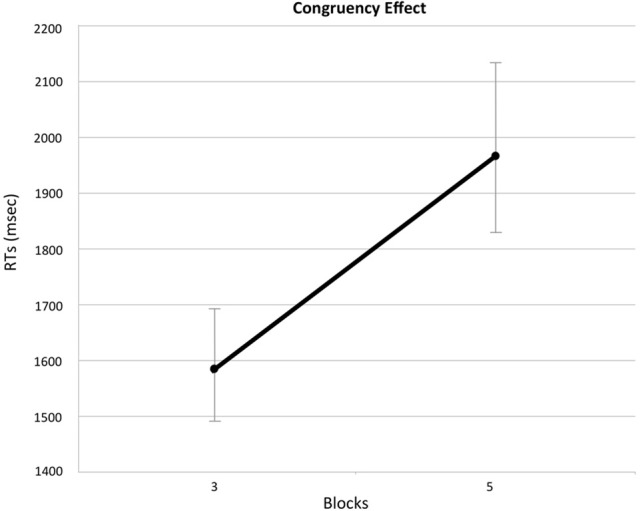
**The effect of Congruency on reaction times (RTs)**. Figure shows the difference in RTs between congruent and incongruent block. Block 3 is the congruent block, whereas Block 5 is the incongruent one. Value of RTs according to the block are represented with their 95% Confidence Interval.

Thus, a significant Congruency effect on RTs emerged (*z* = −25.47, *p* < 0.001; Figure 2). This difference was quantified (ICDiff) and indicated that RTs in the incongruent block were 381.12 ms higher than those in congruent block.

The mean D-IAT for the chance group was 0.61 (*SD* = 0.45, *SE* = 0.083), whereas for the intentional group it was 0.56 (*SD* = 0.32, *SE* = 0.059). Because the mean D-IAT for both groups was positive, this indicates that at a group level, past intentions were correctly identified. The regression model for the D-IAT was not significant, indicating that the Group factor did not significantly influence D-IAT (*p* = 0.60). At an individual level, 57/60 participants' past intentions were correctly detected. Two of the three misclassification cases come from the chance group (Table 3). Thus, the accuracy for the chance group was 93.3% and for the intentional group was 96.7%, leading to an overall accuracy of 95%. The mean D-IAT for the correctly classified participants (positive) was 0.63 (*SD* = 0.35, *SE* = 0.046), which is consistent with studies using aIAT on autobiographical memories but lower than that reported in studies on flashbulb memories (e.g., Curci et al., 2015). On the other hand, the mean D-IAT for the misclassified participants (negative) was −0.25 (*SD* = 0.20, *SE* = 0.12).

**Table 3:** **Classification accuracy**.

**Group**			**Chance**	**Intentional**	**Total**
D-IAT	**+**	n.	28	29	57
		%	93.3	96.7	95
	**−**	n.	2	1	3
		%	6.7	3.3	5
Total			30	30	60

*Number of correctly classified and misclassified participants through D-IAT for each group and for the whole sample with corresponding percentage accuracy. Positive D-IAT values (+) indicate correct classification, while negative values (-) indicate misclassification*.

## Discussion and conclusions

Studies on lies and memory detection have shown that a number of psychophysiological, neural, cognitive, and behavioral measures can be useful to detect real intentions for future actions. To our knowledge none of the previous investigations focused on the detection of intentions behind actions (events) that occurred in the past. Our experiment aimed at investigating whether the analysis of RTs could be useful in detecting past intentions, and in particular at evaluating a variant of the Implicit Association Test, the autobiographical Implicit Association Test (aIAT), as past intentions' detector. We analyzed reaction-times data collected from a sample of 60 participants performing an aIAT task about the intentionality behind a recent meeting with a person they already knew. As different participants reported different meetings, each participant performed a specific tailored aIAT. Our findings show that a significant effect of congruency emerged, if considering all sample data through a Generalized Linear Mixed-Model. That is, in the condition in which true statements and real intentions shared the same motor response, RTs were shorter when compared to those in the condition in which the same response button was shared between true statements and false intentions. This effect is called *compatibility effect*. According to the originators of the IAT (Greenwald et al., 1998), the basis of this effect is the conceptual association between the target and the attribute categories (Kinoshita and Peek-O'Leary, 2005). In the case of the aIAT, it refers to the conceptual association between one of the two versions of the construct under investigation (e.g., intentional meeting) and a logical category (e.g., True). This, in the memory detection perspective, indicates that one of the two versions of the construct under investigation matches with an autobiographical memory and could indicate that information about intentions is encoded in the episodic memories of events. The next step we made was to quantify the amount of compatibility effect for each subject independently through the calculation of the D-IAT Index (Greenwald et al., 2003). Thus, this index allowed us to assess the strength of the association between true statements and real/counterfeit intentionality. On the basis of the sign of the D-IAT Index, as explained in the Methods section, we consequently investigated the accuracy of the aIAT in identifying the real intentions behind each subject's reported event. Results demonstrated that it is possible to identify a past intention through the use of the aIAT with an accuracy of 95% (93.3% sensitivity, 96.7% specificity). Thus, the difference in RTs between the congruent and the incongruent condition—the *compatibility effect*—was able to detect real past intentions.

In a previous study about the detection of future intentions (Agosta et al., 2011a) the neural basis of the *compatibility effect* in the aIAT was investigated using Event-Related Potentials (ERPs). The authors found that Late Positive Component (LPC) had smaller amplitude for the incongruent than for the congruent block. In previous ERP studies, this component was found to be sensitive to conflicting information (Magliero et al., 1984; Doucet and Stelmack, 1999), thus reflecting the allocation of resources between two simultaneous tasks (for reviews, see Johnson, 1986, 1993). In particular, several studies demonstrated that previously learned items elicited larger LPCs than unlearned items over the left parietal scalp in the interval between 500 and 800 ms post-stimulus (e.g., Johnson et al., 1985; Van Petten et al., 1991; Smith, 1993; Johnson, 1995; Wilding et al., 1995), and that this measure was independent from subject's explicit response (e.g., Smith, 1993; Wilding et al., 1995; Johnson et al., 1998). Moreover, this measure was not significantly altered in participants who feigned amnesia (Tardif et al., 2000) or who responded deceptively or carried out some attempt to alter their performance (Johnson et al., 2003). This effect is referred to as episodic memory (EM) effect. Taken together, these results indicate that the parietal EM effect reflects the memory status of the item rather than the status explicitly declared by the subject (Johnson et al., 2003).

More importantly, this enhancement of LPC activity, elicited by stimuli matching episodic memory, appears to be associated with a pattern of shorter RTs (Johnson, 1995; Johnson et al., 1998, 2003) which is basically what is detected by aIAT. These findings support the idea that during the incongruent block, the cognitive load is greater when compared to that during the congruent block. In the aIAT's incongruent condition, participants are requested to associate the real autobiographical memory with false statements. So, because of the familiarity with the stimuli referring to the real autobiographical memory and their automatic association with the concept of “truth,” this task is more cognitively demanding and responses are slower.

The increase in cognitive load is also considered as a key feature for the deceptive process. A growing body of evidence supports the assumption that lying requires a greater cognitive load compared to telling the truth (Zuckerman et al., 1981; Vrij et al., 2006), adding a constant additional time to RTs independently from the complexity of the task or the response method (Sheridan and Flowers, 2010). Thus, some lie detection techniques are based on RTs analysis, starting from the assumption that, while deceiving, a subject would be slower and more error-prone (see Verschuere and De Houwer, 2011, for a review). An example of these techniques is the RT-based version of the Concealed Information Test (CIT; see Verschuere et al., 2011, for a review) previously referred to as the Guilty Knowledge Test (GKT; Lykken, 1959), which has been investigated in laboratory research (e.g., Ben-Shakhar and Elaad, 2003; Meijer et al., 2014) and is also used in criminal investigations in Japan (Osugi, 2011).

The need for a science-based assessment of specific autobiographical memories seems to be increasing in criminal trials, as it operates within a set of rules aimed at establishing overt blameworthy behaviors (*actus reus*) inferred from (guilty) covert mental states. The beliefs, intentions, and motivations of the defendant (*mens rea*) are *post-hoc* inferred from behavior and the reconstruction of contextual events, without the use of any kind of accurate and reliable science-based tool. In the present study we demonstrated that RTs analysis could be useful to accurately detect past intentions.

Starting from this viewpoint, other factors not investigated in this study which could potentially influence aIAT performance in detecting past intentions are, for example, emotional connotation of the reported event and compatibility with our motivations. For instance, consider a hypothetical case of a man who died after being hit by a car while crossing the street on foot. In this case the driver would be accused of vehicular manslaughter (without volition). At this stage, imagine that investigators discovered that the driver was familiar with the victim and that they had had problems in the past (e.g., the driver was the ex-boyfriend of the victim's current girlfriend). Based only on this information, investigators could reconstruct the driver's *mens rea* suggesting that since the defendant had a reason to hate the jaywalker, he should be prosecuted for voluntary manslaughter rather than vehicular manslaughter. In a case like this, investigators may attribute intentionality concerning a crime through an inferential process based on behavioral and circumstance evidence. The critical point in our opinion is that, from this perspective, reason and intention are considered as the same concept. In this context, adding information derived from an accurate tool in the detection of past intentions could be extremely important. Kaasa et al. (2011) faced the issue about accuracy in recalling reasons behind behavior after long delays. In their experiments, participants were first (Time 1) asked to fill out a survey about their music interests and the reasons behind the most recent purchase of a music compact-disc (CD). Then, after 6–12 months (Time 2) a follow-up survey was sent. Finally, a subset of participants was contacted after 6–12 months from Time 2 (Time 3), and they were asked to fill out a third survey. One of the main findings of this study was that only about one-fifth of the participants were able to consistently recall the real reasons why they bought the CD. We think that such an important finding, however, does not undermine our results for at least two reasons: (a) participants in the present study were asked not about the reasons behind their behavior but about the degree of intentionality behind an event they lived and (b) the autobiographical memory on which the task focused was decided by participants and not imposed by experimenters.

This last point could be considered a limitation of this study, in particular if considering the forensic context. Indeed, in criminal trials, a defendant is specifically questioned about an event (characterized by a criminal behavior) he/she lived. Thus, obviously, the autobiographical memory is not freely chosen by the person. Moreover, further studies should consider a group of participants instructed to deceive in order to investigate the possibility to fake the aIAT, already analyzed for different versions of the test (Agosta et al., 2011b).

The results by Kaasa et al. (2011), however, suggest a question regarding the detection of past intentions: if a fortuitous event reflects a vague motivation, how much do we feel implicitly responsible for it and to what extent does this feeling affect our performance on aIAT? A preliminary answer is given by Agosta et al. (2011a) in their work concerning prior intentions about a future action, in which they found good aIAT accuracy both in detecting prior intentions and in distinguishing them from the subject's hopes and expectations. Thus, future research could focus on testing aIAT accuracy in distinguishing between past intentions and motives behind a past event.

The aIAT's original purpose was to identify a true past autobiographical event from an alternative event. Nevertheless, in previous studies it has been tested on the detection of prior intentions (Agosta et al., 2011a) and in detecting reasons behind white lies (Agosta et al., 2013). In the present study, we went a step further by demonstrating that past intentions can be reliably identified using the aIAT with high accuracy (comparable with those previously reported for other versions of aIAT; see Sartori et al., 2008)^1^. Since forensic applications usually need to prove whether there was an intention behind a crime, this study is the first showing that RTs-based memory detection techniques can be useful in the detection of past intentions.

### Conflict of interest statement

The authors declare that the research was conducted in the absence of any commercial or financial relationships that could be construed as a potential conflict of interest.

## Footnotes

^1^Several independent research groups confirmed the accuracy level of the aIAT (e.g., Hu and Rosenfeld, 2012; Hu et al., 2012; Freng and Kehn, 2013). Only a study by Vargo and Petroczi (2013) reported a lower accuracy; however, these authors used a brief version of the aIAT together with negative sentences, which have to be avoided in aIAT according to its authors' suggestions (Agosta and Sartori, 2013). In her master's thesis, Dr. Eleonora Zecchinato collected data from 103 volunteer participants using original aIAT with no negative statements, and she demonstrated that the accuracy was higher (Zecchinato, 2014).
